# Advances in targeting EGFR allosteric site as anti-NSCLC therapy to overcome the drug resistance

**DOI:** 10.1007/s43440-020-00131-0

**Published:** 2020-07-14

**Authors:** Swastika Maity, K. Sreedhara Ranganath Pai, Yogendra Nayak

**Affiliations:** Department of Pharmacology, Manipal College of Pharmaceutical Sciences, Manipal Academy of Higher Education, Manipal, Karnataka 576104 India

**Keywords:** Allosteric site, EGFR, NSCLC, Fourth-generation lung cancer therapy, Anti-NSCLC agent

## Abstract

**Background:**

The epidermal growth factor receptor (EGFR) inhibitors represent the first-line therapy regimen for non-small cell lung cancer (NSCLC). Most of these inhibitors target the ATP-site to stop the aggressive development of NSCLC. Stabilization of the ATP-binding on EGFR is difficult due to autophosphorylation of the EGFR domain. This leads to activation of nonintrinsic influence of the tumor microenvironment and expression of anti-apoptotic pathways and drug resistance.

**Methods:**

The NSCLC related literature search was carried out using online databases such as Scopus, Web of Sciences, PubMed, Protein Data Bank and UniPort for the last ten years and selected articles are referred for discussion in this review.

**Results:**

To overcome the problem of mutations in NSCLC, the allosteric site of EGFR was targeted, which shows significant therapeutic outcome without causing resistance. Compounds like EAI001, EAI045 JBJ-04-125-02, DDC4002 and a series of small molecules with an affinity towards the EGFR allosteric site are reported and are under the investigational stage. These compounds are categorized under fourth-generation anti-NSCLC agents.

**Conclusion:**

Composition of this review highlights the advantage of inhibiting allosteric site in the EGFRTK receptor domains and presents a comparative analysis of the new fourth-generation anti-NSCLC agents to overcome the drug resistance.

## Introduction

Epidermal growth factor receptor (EGFR) belongs to the family of protein kinase (PK) widely distributed throughout the human body. In the normal cell, EGFR is responsible for regulating the mTOR-serine/threonine-protein kinase pathway and maintenance of down-stream signaling pathway; which overall maintains the growth of hepatic cells [1]. Any malignant transformation in EGFR can cause lung cancer propagation [2]. Hence, pharmacotherapy has been developed to inhibit EGFR, and they have become the main class of drugs in the treatment of NSCLC [3]. Among the lung cancers, 80–85% belong to NSCLC [4]. The WHO reports says the lung cancer deaths highest among the cancer deaths, and each day, 422 deaths are caused globally due to NSCLC [5, 6]. Till 1980s, NSCLC cases were recorded more in men than women, but in the past decade, there was an increase incidences in women compared to men [6]. One in two million cases of NSCLC were in the pediatric population, and 6% of them are asymptomatic. The mortality rate of NSCLC is 51% in the pediatric population, with a survival rate of only 26% [7]. However, the occurrence of pediatric lung cancer is 0.049 per 100,000 population [8]. Also, the mortality rate of NSCLC is double in the African-American male as opposed to Asian-Americans, which accounts to have the least number of cases. African-American men are 20% more likely to develop lung cancer as compared to Caucasian men. The disease prognosis rate is 10% lower in African-American women than in Caucasian women. The racial and ethnic disparities are mainly due to differences in smoking prevalence, a higher probability of diagnosis only during advanced stages of NSCLC in African-Americans, Africans and lower rates of resection [9]. Hungary followed by Serbia, then France are reported to have the highest morbidity and mortality rate of NSCLC with 56.7, 49.8 and 42.3 age standard rate per 1,00,000 [10]. This disease has been showing varying trends across different age groups, gender, and races. All these factors make the research for NSCLC difficult due to its varying pattern. Therefore, to develop an improved pharmacotherapy, it is crucial to understand the pathophysiology of the disease along with its epigenetic mechanisms.

In the past few years, NSCLC has proven to be a serious economic burden, amounting to approximately US $2.6 billion being used for drug development and capitation with 8.6% cost inflation each year for the same [11]. Besides, oncology drugs receive priority review by the Food and Drug Administration (FDA), the drug discovery and development rate for NSCLC have remained low [12]. In the past three decades, out of 591, only 60 anti-NSCLC drugs have been reached to phase-3. The phase transition probability of NCSLC drugs is 8.0%, with a 92% attrition rate [3]. Thus, drug discovery and development is lowest for NSCLC as compared to other types of cancer. This data is alarming since the disease with the highest mortality rate among cancers also has the highest attrition rate in terms of drug discovery and development.

The current treatment of NSCLC involves surgery, radiation, chemotherapy, and targeted therapy depending on the cancer stage. More than 40% of newly diagnosed lung cancers will be in stage IV, and cytotoxic combination chemotherapy is recommended as first-line therapy. The generally used chemotherapy includes platinum (cisplatin or carboplatin) in combination with paclitaxel, gemcitabine, docetaxel, vinorelbine, irinotecan, or pemetrexed. The drug paclitaxel also called as taxol, docetaxel a semi-synthetic taxol, vinorelbine a semi-synthetic vinca alkaloid act upon microtubule and polymerases or stabilizes it to inhibit the cell division. The drug gemcitabine inhibits the DNA synthesis required for cell divisions. The irinotecan a derivative of camptothecin inhibits topoisomerase I. The oreetrexed a antimetabolite antifolate agent used in combination for NSCLC. But, none of the single-drug regimens is significantly superior over a combination of two or more drugs [13]. With the advancement in personalized medicine, the targeted drugs have been improved survival in patients with NSCLC. The EGFR inhibitors such as erlotinib, gefitinib, afatinib, dacomitinib, osimertinib and rociletinib currently have their major role in the therapy of NSCLC. Similarly, anaplastic lymphoma kinase (ALK), ROS1/RET, MET, BRAF, HER2, KRAS, AKT are other validated targets in NSCLC therapy [14].

The enzyme inhibition property of the EGFR-TK allosteric site targeting drugs results in a functional impact on the cell-signaling cascade [15]. The novelty in the allosteric site has driven the attention of researchers and is considered as the most potential target for the development of anti-NSCLC agents. Hence, this review outlines the role of EGFR allosteric sites inhibition which converges on the TK-receptor ligand interaction.

## Literature search methodology

A literature search was conducted using online databases such as Scopus, Web of Sciences, PubMed, Protein Data Bank and Uniport for the last 10 years. The keywords used for the literature search were “lung cancer”, “NSCLC therapy”, “allosteric site EGFR”, “EGFR inhibitors”, “fourth-generation NSCLC drugs”, “anti-NSCLC agent”, “drawbacks EGFR inhibitor”. The literature search aimed to get all important information and research data, which can enhance the knowledge of allosteric site inhibition as a potential target in EGFR TK. The selected articles are referred for discussion in this review.

## Current therapy for NSCLC and their limitations

### Combination chemotherapy

The intravenous platinum doublet-agents are the first-line chemotherapy for NSCLC. Platinum triplet therapy is used as a substitute platinum-doublets. Generally, chemotherapy regimen includes a platinum-based combination with third-generation EGFR inhibitors or pemetrexed, bevacizumab, and ceritinib or crizotinib. The therapy is successful in providing therapeutic relief to only 30%. The acquired resistance development in patients or, development of tumour progression after 5 months of treatment is the main reason for poor efficacy. The platinum-based combination of gemcitabine, taxane and vinca alkaloids reported a response rate (RR) of 17–32%, median progression-free survival (PFS) 3.1–5.5 months and median overall survival (OS) between 7.4 and 9.9 months. Platinum-based combination with cisplatin and carboplatin showed RR to be 25 and 28% respectively, and OS calculated to be 9.8 months and 8.2 months respectively [16]. The main mechanism of action of chemotherapy is the intrastrand crosslink due to platinum-induced lesions. This intrastrand crosslink is highly cytotoxic due to blockade of the replication and transcription process. This crosslinking triggers the DNA damage in G_0_ and G_1_ phases of the cell cycle (Table 1). Even though the platinum-complexes are categorized under, the first-line therapy majority of the patients receiving the chemotherapy showed platinum resistance [17].

**Table 1:** Summary of anti-NSCLC agents

Sr. no.	NSCLC drug classification/therapy name	Name of drugs	Mechanism of action	Inhibitory site in EGFR	Drawback	References
1.	First-generation NSCLC drugs	Erlotinib, gefitinib	Binds to the ATP binding site of EGFR which leads to inhibition of auto-phosphorylation in kinase domain, further leading to inhibition of dimer formation of the kinase	ATP binding site of EGFR and shows interaction at The790	(1) Resistance development at ATP binding site (2) It cannot completely inhibit auto-phosphorylation, which leads to instability in the kinase domain leading to development of NSCLC	[20, 21, 39]
2.	Second generation NSCLC drugs	Afatinib, dacomitinib	These drugs inhibit the enzymes, which lead to the activation of T790M by irreversible binding to the EGFR kinase domain. The inhibition mainly takes place due to the structural similarity of drugs to target enzymes	Inactive site of EGFR	(1) The drugs only recognizes the dimer complex of kinase but not the monomeric state. So, the drugs are not specific for therapy of NSCLC	[25, 26]
3.	Third generation NSCLC drugs	Osimertinib, rociletinib	Due to the pyrimidine structure of the drugs, it covalently binds to ATP site of EGFR domain and selectively blocks the overexpression of T790M	ATP site of EGFR	(1) Development of C797S mutation which lead to resistance development for the drugs	[29, 30]
4.	Combination therapy	Docetaxel + pemetrexed	The drugs leads to enzymatic inhibition in the pathway of cell proliferation and apoptosis	Enzymatic inhibition of the NSCLC pathway	(1) Skin rash (2) Diarrhea (3) Hair loss (4) Neutropenia	[19]
5.	Salvage therapy	Cetuximab + docetaxel or pemetrexed	A synergistic effect was observed which makes the combination therapy more potent and binds strongly to the ATP site of EGFR. This inhibits auto-phosphorylation of the kinase domain	ATP binding site of EGFR	(1) Response to the therapy is observed after 6 months or more	[31]
6.	Fourth-generation NSCLC drugs	EAI001, EAI045	Binds to the allosteric site of EGFR along with ATP that leads to inhibition of auto-phosphorylation and dimer formation of the kinase domain. Thus, giving stability to overall EGFR and stops the resistance development problem	Allosteric site of EGFR	No side effects or adverse drug effect reported so far	[46, 47]
7.	First-generation allosteric EGFR inhibitor/fourth-generation NSCLC drug	JBJ-04-125-02, DDC4002	Binds to the allosteric site of EGFR along with ATP that leads to inhibition of cell proliferation and arrest of EGFR L858R/T790M/C797S signalling	Allosteric site of EGFR	(1) Oral bioavailability is low (2) Long-term treatment lead to drug accumulation	[50, 51]
8.	Small affinity allosteric EGFR inhibitor		Binds to the allosteric site of EGFR along with ATP that leads to inhibition of auto-phosphorylation and dimer formation of the kinase domain. Thus, giving stability to overall EGFR and stops the resistance development problem	Allosteric site of EGFR		[49]

Different categories of anti-NSCLC agents with their mechanism of action along with the target site of EGFR. The table briefly highlights the drawbacks of each category of anti-NSCLC agents

*NSCLC* non-small cell lung cancer, *EGFR* epidermal growth factor receptor, *ATP* adenosine triphosphate *DNA* deoxyribose nucleic acid

Docetaxel is an anti-mitotic chemotherapeutic agent, used mainly for the treatment of ovarian, breast cancer, and NSCLC. Docetaxel reversibly binds to tubulin with high affinity in a 1:1 stoichiometric ratio. Pemetrexed is useful in combination with cisplatin for the treatment of patients with malignant pleural mesothelioma, which is either unresectable or not eligible candidates for curative surgery. This is due to the inhibition property of thymidylate synthase, dihydrofolate reductase and glycinamide ribonucleotide formyltransferase of pemetrexed, which makes it antifolate agent [18]. The combination therapy of docetaxel and pemetrexed was able to slow down the aggressive nature of NSCLC by inhibition of cell proliferation pathway (Table 1). Due to high side effects like skin rash, diarrhea, neutropenia, and hair loss, this combination therapy is not preferred by the health care professionals [19].

### First-generation EGFR drugs for NSCLC

The first-generation EGFR drugs, namely gefitinib and erlotinib, are approved for the treatment of NSCLC in 2003 and 2004 by US-FDA. These drugs are EGFR-ATP binding inhibitors. Their efficacy failed due to secondary mutation. Threonine-790 (T790M) is tagged to be the “gatekeeper residue” for the inhibition of activation of the ATP site in EGFR [20]. It was also understood that higher the ATP site affinity more will be the chances of T790M mutation in NSCLC and drug resistance. One of the reasons for the development of NSCLC is exon 19 multi-nucleotide frame deletion, which leads to the dimerization of TK complex of EGFR [21]. This dimerization leads to the elimination of four amino acid sequences, Leu–Arg–Glu–Ala. Thus the mutated TK-EGFR dimer auto-activate even in the absence of ligand [22]. Few cases of NSCLC mutations showed that single nucleotide substitution in exon 21 could also cause resistance development in the ATP binding site. Mutation in the exon 21 region results in the replacement of arginine for leucine at L858R [23]. Yet, the true cause of resistance development remains uncertain (Table 1). Due to many such factors, the first-generation drugs are unsuccessful.

### Second-generation EGFR drugs

The first generation gefitinib use was restricted in 2005 and later withdrawn in 2012 by US-FDA on the basis of negative results in phase-III trials. But there are opinions that they can still have their role in spite of third-generation drugs [24]. To overcome the drawbacks of first-generation drugs, the anilinoquinazoline derivatives namely, afatinib and dacomitinib, were introduced. These drugs are approved by US-FDA later in 2010–2011. These drugs were reported to be multitarget TK inhibitors for anti-NSCLC therapy [25]. The main mechanism of action of the second-generation drugs is enzymatic inhibition of the mutant EGFR-T790M by irreversibly binding to the kinase domain [26]. The second-generation drugs recognize only inactive T790M; therefore, it also targets the inactive site of the kinase domain. However, the inactive site only recognizes the kinase dimer complex of the kinase. The dimer state occurs only after the auto-phosphorylation of the TK domain (Table 1). One of the drawbacks of second-generation drugs that it fails to target the monomeric state of the kinase domain. Hence, these groups of drugs are not completely suitable for inhibition of NSCLC.

### Third-generation EGFR drugs

The EGFR secondary mutation or T790M mutation is the major reason for the resistance development in the first and second-generation drugs [27]. Osimertinib and rociletinib are pyrimidines classified under third-generation anti-EGRF drugs. Osimertinib is an irreversible binder and due to its pyrimidine structure, covalently binds with the ATP site of the EGFR domain. This makes it a selective blocker for EGFR activated T790M resistant mutation. The resistance development due to the mutation in the gatekeeper residue (T790M) [28]. Osimertinib, which is an irreversible EGFR inhibitor, covalently interacts with Cys797 targeting the T790M mutant in EGFR. But Cys797 mutation to serine (C797S) adversely affects the therapeutic benefits of osimertinib. However, the third generation drug showed C797S mutation, which leads to the development of resistance in patients [29] (Table 1). The objective is to stabilize the PK activation by inhibition of the rapid interconversion between the monomeric state (inactive form) and the diametric state (active form) of the TK receptor [30]. Thus the ATP binding site and the inactive site of the kinase domain are not suitable as target for developing pharmacotherapy of NSCLC. Therefore, the identification of new targets is imperative for drug development.

### Salvage therapy

Zhang et al. (2015) studied the combination of cetuximab with other anti-cancer drugs like docetaxel and pemetrexed individually. Even though the patient did not show complete response to the combination therapy, the objective response rate (ORR) was 11.8%, compared to the disease control rate (DCR) 50% and disease progression rate (DPR) 50%. This therapy regimen identified to be as salvage therapy when all other drugs fail to respond. One of the major drawbacks was that the therapy showed the response only to the EGFR-TK pathway and had a delayed response of over 6 months (Table 1). Thus, combination therapy is not completely successful due to delayed dose–response. Even though there is progress in the development of treatment for NSCLC, the prognosis remained poor [31].

## Role of EGFR in NSCLC and gatekeeper mutations

**Fig. 1:**
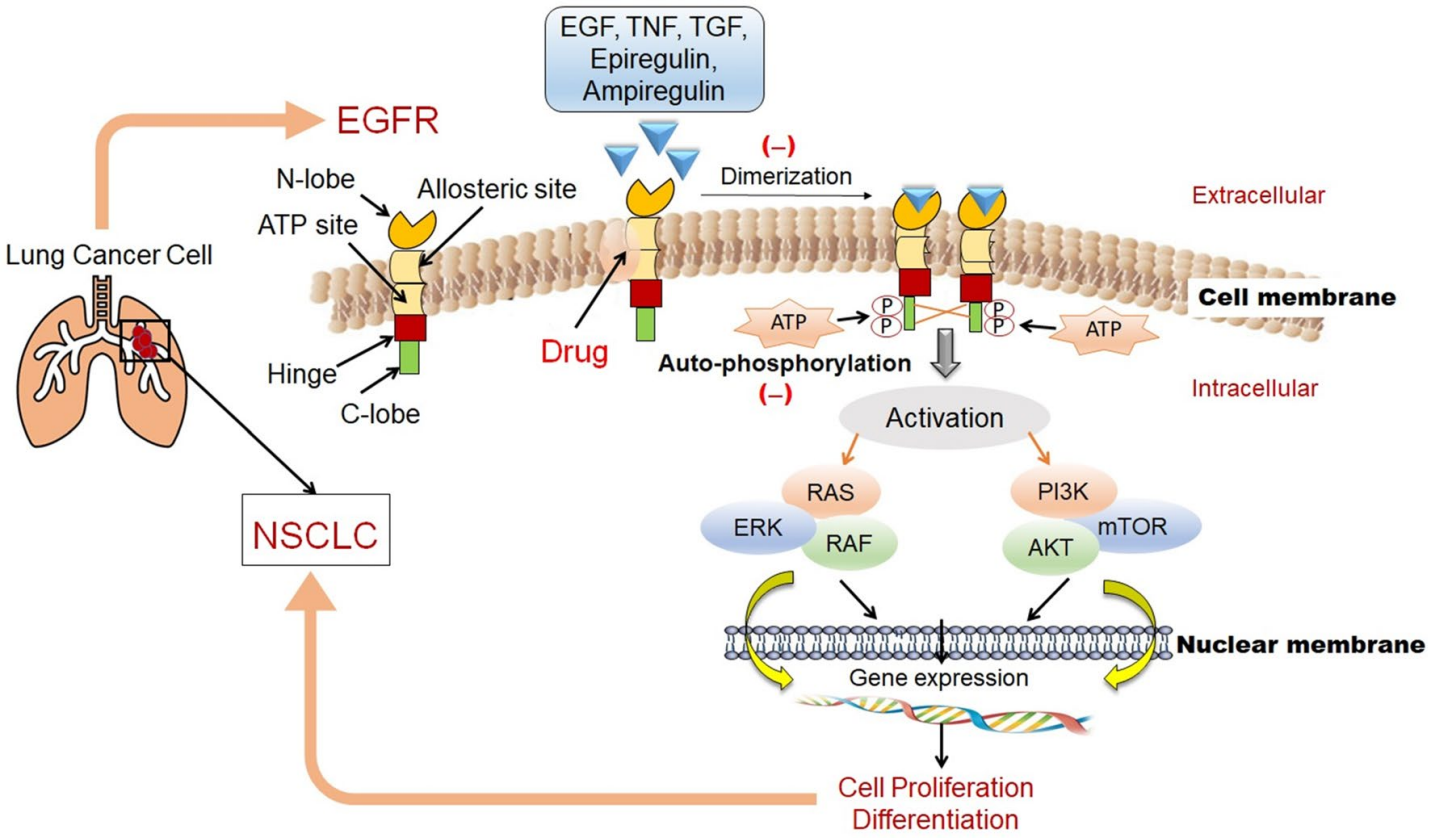
Role of EGFR in NSCLC. The diagram highlights the important markers like RAS, RAF, ERK, PI3K, AKT, and mTOR of the disease and the mechanism of action of drugs against NSCLC (details refer to text and ref. 33–35)

EGFR targeting drugs are recognized to be the drug of choice for the treatment of NSCLC [32]. First EGFR drug ZD1839 was discovered in 1998 with a 14-day regimen of therapy [33]. In 2004, EGFR was linked to mutations, and researchers started exploring all the possible targets in EGFR and other gatekeeper mutations to tackle NSCLC. The acquired resistance mechanism in the first and second generation of EGFR drug was observed. Based on these observations, the current strategy for NSCLC research shifted to targeting EGFR-TK mutation [34]. When ligand interacts with TK, conformational changes occur in the receptor, which leads to auto-phosphorylation of complex formed which further initiates signal transduction cascade. The auto-phosphorylation is due to the catalytic reaction, which converts the ATP and the l-tyrosyl in the EGFR to ADP and *O*-phospho-l-tyrosyl [35]. This cascade is responsible for the activation in the cell proliferation cycle in NSCLC through RAS/RAF/mitogen-activated protein kinase (MAPK) pathway, which is important for the regulation of cell survival. The cascade indirectly activates phosphatidylinositol-3-kinase (PI3K)/AKT/mammalian target of rapamycin (mTOR), which causes inhibition of apoptosis and cancerous growth [35] (Fig. 1).

**Fig. 2:**
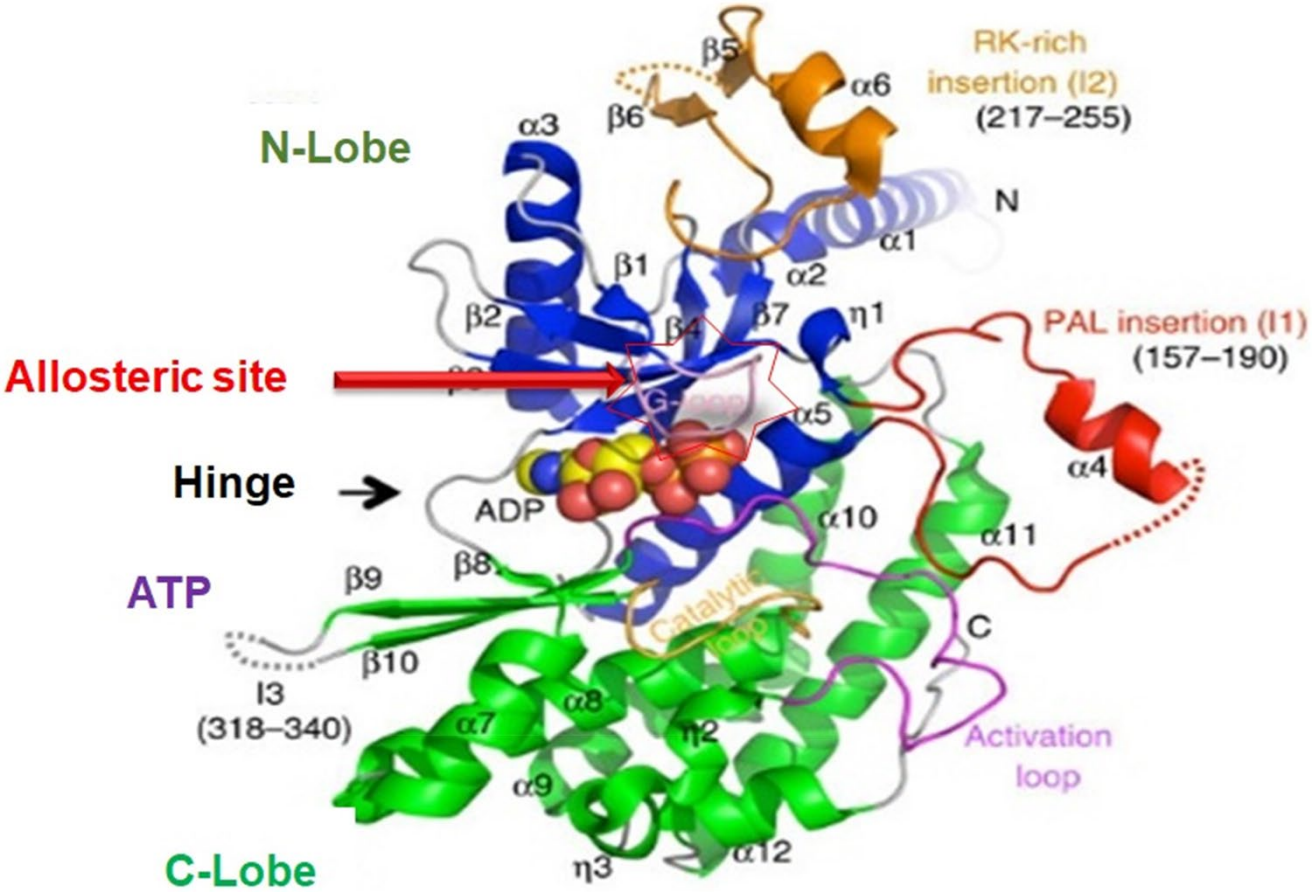
Structure of EGFR TK. The diagram highlight the location of potential druggable target sites like ATP binding site and allosteric site in the EGFR domain. The N-lobe, C-lobe, allosteric site, hinge region facilitates ligand-protein interaction (Read text and ref. 38)

The kinase structure has five major regions, namely, N-lobe, C-lobe, ATP site, hinge region and the allosteric site (Fig. 2). N-lobe has a short amino acid hand with five anti-parallel β-sheets and one α-helix. N-lobe interacts with the C-lobe via the ATP binding site, which is a highly reactive site of the kinase domain. ATP-site binds both the lobes by a thin hinge region [36]. Ligand interacts with the N-lobe that leads to an extension of the α-helix hand. The α-helix region allows the ligand to bind to the extended arm. The ligand-binding leads to conformational changes in the kinase structure and activates the ATP site for auto-phosphorylation. Once the phosphorylation starts, the C-lobe shrinks towards the N-lobe and the kinase receptor are activated [37]. The ATP site is a highly reactive and unstable site due to continuous auto-phosphorylation (Fig. 2).

Drugs developed so far for lung cancer are protein kinase antagonist, which are a steady-state competitive inhibitor [25]. The ATP binding site of PK was not targeted because of structural similarity and faulty hydrogen bond in the site. Thus, the ATP binding site is highly unstable. Therefore, the ATP site antagonist exhibits a large number of side effects. Allosteric site is hidden inside the hinge region and is difficult to target, which makes the allosteric site highly stable and difficult to target [38].

The distinguishing feature of EGFR is the prolonged activation of auto-phosphorylation. Major ligands that interact with TK and lead to dimerization of the TK complex are an epidermal growth factor (EGF), tumor necrosis factor (TNF), transforming growth factor α (TGF-α), epiregulin, and amphiregulin. The EGFR family attains consecutive kinase activation even in the absence of phosphorylation and diametric state attenuation. Hence, the receptor remains inactive even in the absence of ligand interaction or activation factor. Once it is activated, phosphorylation takes place without the involvement of ligand. This phosphorylation leads to prolonged dimerization of the TK-domain. The currently available drugs interact with the receptor partially, which further leads to steric hindrance between the drug and receptor, leading to a decrease in therapeutic efficacy. This prolonged conformational change in the TK receptor affects the drug interactions with the receptor. The drug binds weakly to the receptor, which further decreases the overall therapeutic efficacy. Hence, to achieve the therapeutic index, the patients are subjected to a high dose regimen over time; which subsequently leads to the development of resistance and hepatotoxicity [39]. Thus the hepatotoxicity is the major drawback of NSCLC therapy targeting EGFR-TK receptors. The TK-receptor enables transmembrane signalling, whereas TK within the cell helps in signal transduction to the nucleus. Phosphorylation of proteins by kinases is an important mechanism in communicating signals within the cell and regulating the cellular activity, such as cell division and cell proliferation.

## EGFR-allosteric site as a potential target and its advantages

The first, second and third-generation drugs have failed to produce therapeutic benefit due to EGFR mutations. Targeting the allosteric site has gained attention for the therapeutic relief for many indications [40]. The vast range of benefits targeting allosteric site hints about its potential to serve as a strong target site for diseases with high mortality rates in NSCLC. The phenomenon of allostery is when an effector/ligand/molecule binds to one side of the molecule it causes conformational modification to another site of the protein. This ultimately leads to dynamic or shape changes in the protein complex. Ligand binding to the allosteric site does not cause any conformational changes in the active site [40, 41].

**Fig. 3:**
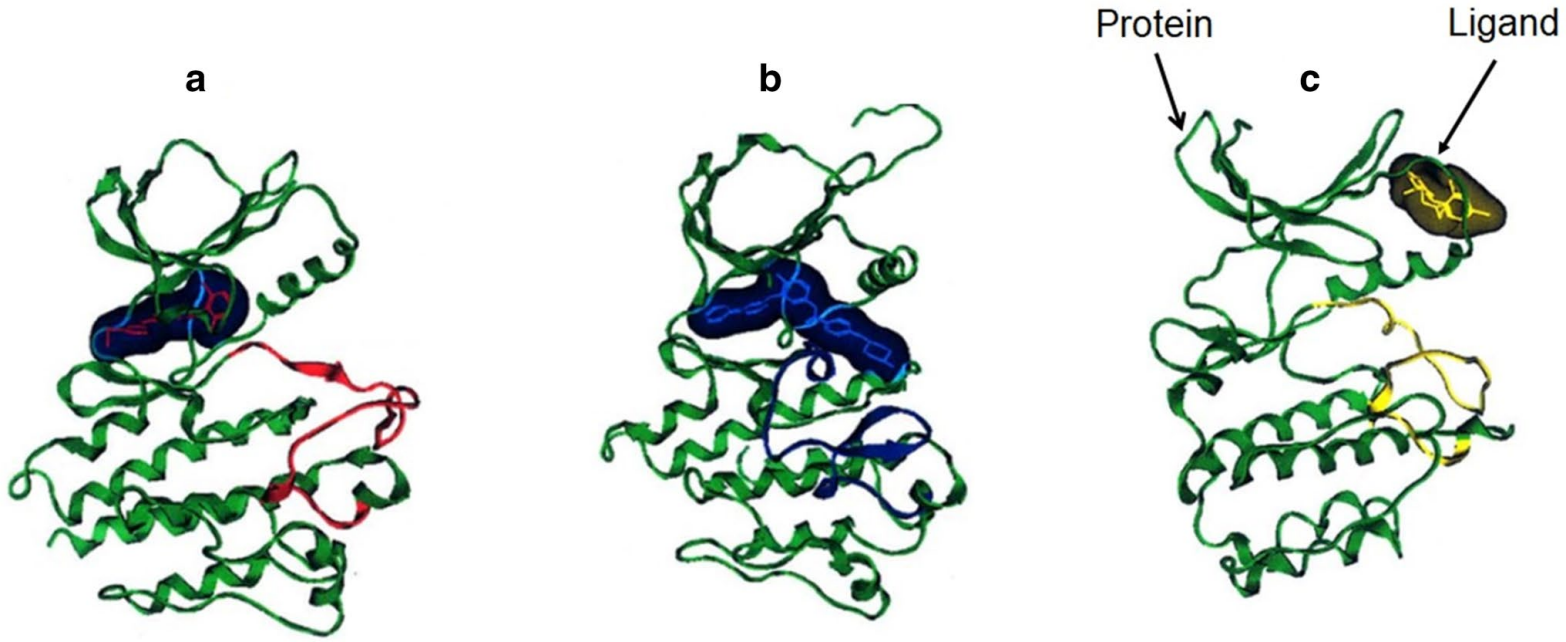
Actions of ligand or inhibitors on binding site in TK receptor. **a** Shows the ligand interaction with the inactive site, which leads to blockade of this site. **b** Shows the interaction of the ligand with the active site, which leads to inhibition of only active site of TK and **c** represents the allosteric inhibition due to ligand interaction which not only inhibits this site but also blocks the activity of ATP site

A TK receptor has three binding sites, namely, ATP competitive site, inactive site and allosteric site. The most of the currently available drugs act on ATP binding site and there are no drugs which act on inactive site. Inactive site of the receptor is the region where the protein does not interact with ligand or drugs. This region is not directly involved with the pharmacotherapeutic response. The binding of effector to the allosteric site on the protein leads to conformational changes at the active or binding site, which regulates protein activity [42]. Allosteric ligands/effectors or drugs are of two types (1) non-covalent and (2) covalent binding agents. Allosteric changes are initiated by mutation, binding events or post-translational modification. Low dose allosteric drugs are mostly non-covalent binders and therefore, cause low toxicity. This is because, in the absence of drug, the energy barrier between the two states is high. Hence, it will be switching back to the active conformation. Allosteric regulation is divided into three separate sites (1) substrate site, (2) allosteric site and (3) functional site or active site. This property of allostery can help the researchers to formulate low drug doses which ultimately reduce the adverse effects of the drugs. The allostery curtails the effect on gene signal regulation, cellular apoptosis, receptor trafficking, and signal transmission. The allosteric inhibition could be analyzed based on three characteristics, namely, (1) selectivity, (2) potency and (3) effectiveness. Targeting the allosteric site leads to the stable conformation of the complex formation with protein, which affects both the efficacy and binding of the primary ligand. This makes the protein less active to the ligand-complex formation or shows neutral functionality during ligand interaction. Therefore, allosteric sites have the potential to inhibit or completely cease signal-induction cascade. Besides, this site is conserved or stable (Fig. 3). Thus, targeting allosteric site can be utilized for inhibition of cellular proliferation in cancerous conditions. Therefore, allosteric inhibitors can have high specificity. The positive allosteric modulators increase the sensitivity of the target receptor by lowering the overall threshold of the cellular pathway [43].

Thus, the novel properties and characteristics of the allosteric site help to overcome the problem of “undruggable” proteins and reduces the chances of developing side effects and adverse effects of the therapy. If such proteins have transient allosteric pockets, then the proteins can be explored for its direct or indirect activity. Targeting the allosteric site can lead to collective therapy development and helps explore the pharmacology of such proteins. For this reason, the allosteric site retains the potential to become the novel target for the development of anti-NSCLC agent [26].

**Fig. 4:**
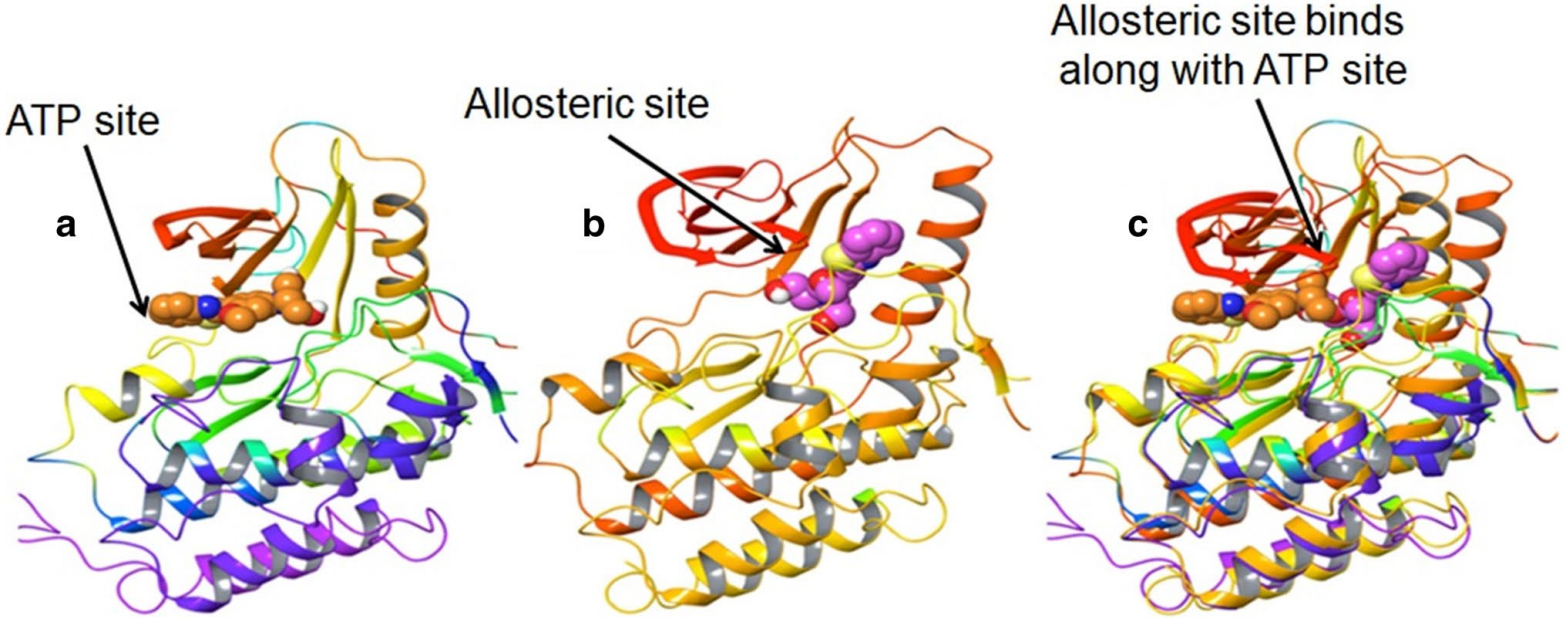
Inhibition sites on EGFR. Read the text for details and ref. [41]

**Fig. 5:**
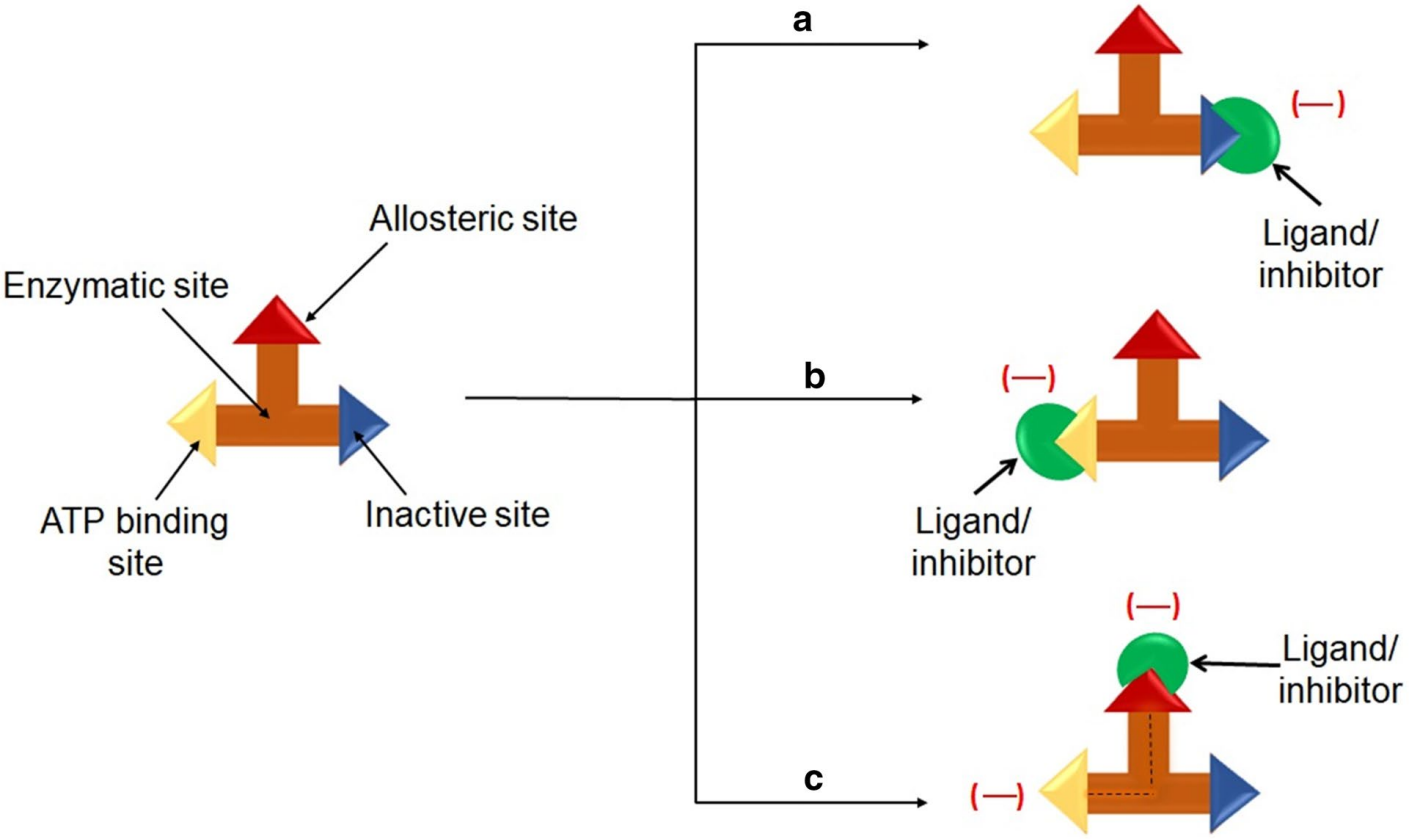
EGFR-TK receptor binding sites. ATP binding sites on EGFR, diagram **c** represents the superimposition of **a** and **b**. Diagram **c** demonstrates how the allosteric site is associated with the ATP site when the drug/inhibitor/ligand targets the EGFR. The allosteric site has the potential to stabilize the whole kinase domain by binding along with ATP [read the text for details and ref. (44, 45)]

When allosteric site inhibitors bind with the ATP site but do not interact with the hinge region, this leads to blockade in kinase activity without displacing ATP (Fig. 4). Further, this leads to inhibition of auto-phosphorylation and the conformational change in the kinase domain is also inhibited, which facilitates in achieving equilibrium [44]. The allosteric site can be targeted to form a stable interaction with the inhibitory drug. In the case of drug-target interaction, it is the most stable bond and thus can achieve conformational equilibrium (Fig. 5). This could solve concerns of resistance development which is seen in current therapy due to the lack of interaction of drugs with the TK receptor.

## Fourth-generation agent targeting EGFR and their structures

Jia et al. [46] and Zhao et al. [47] identified two compounds individually (EAI045 and EAI001), showed the potential to overcome the resistance. These novel compounds were synthesized by screening over 2.5 million compound library to see allosteric site interaction property. These compounds were reported to inhibit the ATP activity and stop auto-phosphorylation, thus, inhibiting T790M mutation. When a ligand binds to the allosteric site, C-helix leads to outward displacement in an inactive conformation. This provides overall stability to the TK domain. These compounds were reported to inhibit L858R/T790M mutation. Both the compounds are non-ATP competitive and mutant sensitive. Both the compounds were analyzed for its sensitivity against 250 types of protein kinase. It was found that these compounds are only sensitive to EGFR [46, 47] (the relevant details are given in Table 1).

EAI001 and EAI045 bind to EGFR T790M/C797S/V948R that lies deep inside the EGFR towards the ATP binding site and C-helix. These compounds comprise of a thiazole aromatic ring, isoindoline-1-one and *para*-fluorophenol moiety, which interacts with Met790 and Phe856 side-chain. The compounds, EAI001 and EAI045, showed inhibitory activity due to hydrophobic interaction with amino acid Ile759, Leu747, Leu788, Leu777 and Met766. These compounds are polar due to the interaction of its nitrogen atom in the amide moiety with Asp 855 in the allosteric site of EGFR. Compound EAI001 has different confirmation as compared to EAI045 due to its interaction with gene T790M/V948R and ANP, which hinders the free movement of the Met790 side chain (Table 2). The most favourable interaction between the allosteric site and fourth-generation anti-NSCLC is mainly due to the activity of thiazole with the target site [46].

**Table 2:**
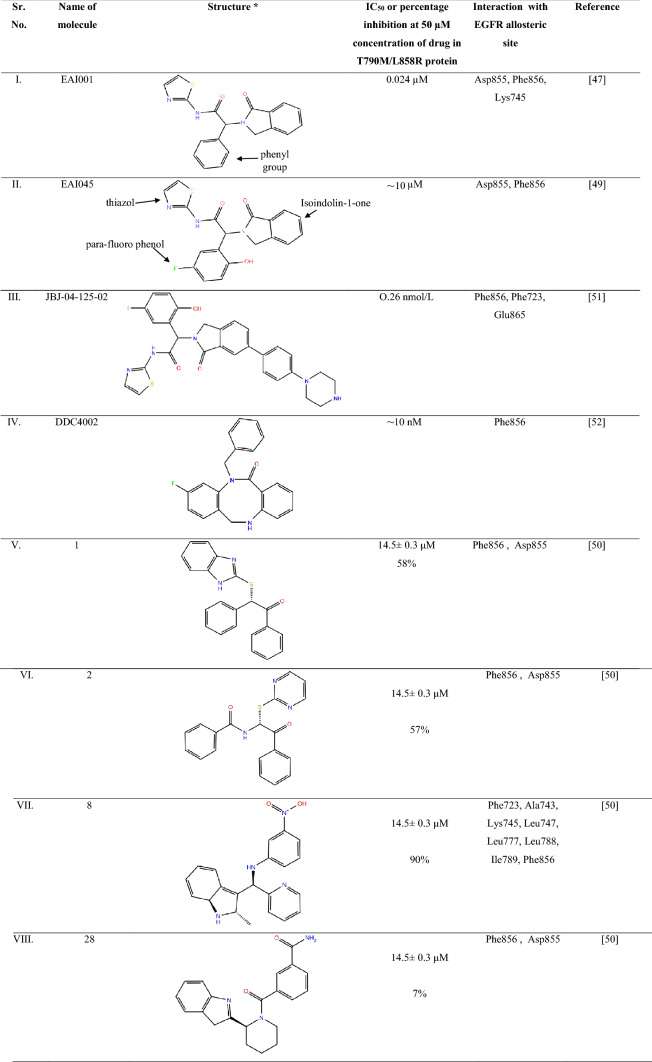

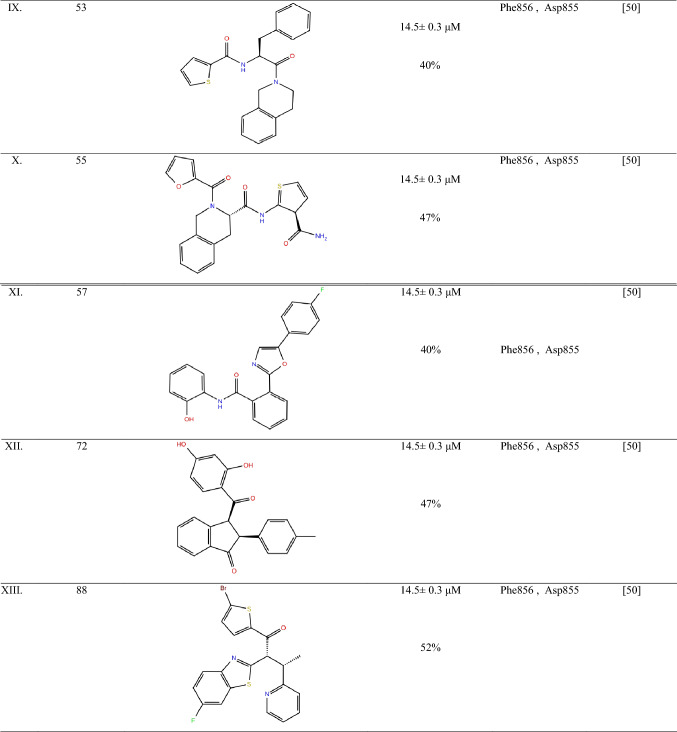
Summary of the EGFR allosteric inhibitors

EGFR allosteric inhibitors with their chemical structures are represented in the table highlighting their IC_50_ value or percentage inhibition at 50 µM concentration of drug in T790M/L858R protein. The table also focuses on the main interaction site of the ligand with the allosteric site of EGFR. All the compounds are in pre-clinical stage of study. All the chemical structures were drawn in chemdraw referring to respective literature source

*EGFR* epidermal growth factor receptor, *Asp* aspartic acid, *Phe* phenylalanine, *Lys* lysine, *Ala* alanine, *Leu* leucine, *Ile* isoleucine

## Secondary structure assessment of EGFR EAI045 and EAI001

EAI045 have two chains (chain A and B) of which 38% helix and 14% is β-sheets. EAI045 has three residues, namely ANP A1102, which is ATP moiety; 57N 1103, which is an inhibitory ligand and MGA 1101, which is magnesium ion moiety [46]. The whole protein entity is composed of 331 amino acids, ranging from residue 695 to 1022 [46]. EAI001 has only one chain (A) of which 28% is composed of the helix, and 14% is the β-plated sheet, which comprises 355 amino acids ranging from residue 675 to 1022 [47]. EAI001 has one inhibitory residue, namely 9LLA 1101 [42]. The figure states the residue sequence of both the compound along with this interaction site with the protein in the allosteric site of EGFR. These interaction sites are the main target for the inhibitors to bind along with the protein and can be a potential target for the identification of new moieties against EGFR mutation [47, 48].

## Difference between EAI001 and EAI045

EAI045 binds tighter to the allosteric site of EGFR due to the presence of *para*-fluorophenyl in place of phenyl group at the ortho position thiazole ring of EAI001 (Table 1). This group make the compound tightly bind to the allosteric site of EGFR at Phe856 increases the binding capacity and increases its specificity to T790M inhibition. Also, the aromatic moiety of compound EAI045; thiazole interacts with Met790, phenyl of *para*-fluorophenol interacts with Phe 856 side-chain and isoindoline-1-one interacts with Ile759, Leu747, Leu777 and Met766 side chain of the allosteric site of EGFR [46, 47]. This leads to an approximately 1000-fold increase in selectivity to wild type EGFR at 1.0 mM ATP. It also showed high interaction with Lys745, which is the methionine gatekeeper residue in the receptor [47–49]. Therefore, the activity of EAI001 is less specific to the allosteric site of EGFR and EAI045 has more potency for inhibition of the allosteric pocket of EGFR in case of NSCLC. Overall, EAI045 can be a potential target to identify new interaction against NSCLC.

## Limitations of the fourth-generation EGFR inhibitors

EAI045 is not effective in monotherapy one reason could be due to overall limitation in the information available for predictive biomarker and insufficient knowledge about the checkpoint inhibitors in case of NSCLC [48, 49]. EAI045, when administered along with cetuximab, shows a synergistic effect by blocking the EGFR dimerization. Cetuximab is an ATP competitive inhibitor of EGFR. Biding of cetuximab ultimately leads to stability in the structure of EGFR domain. The mechanism of action of this combination therapy was studied in the mouse model of lung cancer where it showed that the drug regimen inhibits the L858R/T790M and C797S resistance mutation. This eventually stabilizes the structure by inhibition of auto-phosphorylation. However, the combination therapy shows high bioavailability and efficacy when administered orally or intravenously [48]. The combination therapy of the fourth generation kinases targets the allosteric site by converting the inhibitor-resistant receiver-population into a monomeric form that is particularly sensitive to EAI045 [49]. EAI001 cannot bind to the wild type TK due to its steric clash with Leu 858 and Leu 861 in the N-terminal portion of the active loop. Due to this reason, EAI001 cannot inhibit exon 19 deletion mutation [46].

## JBJ-04-125-02 against EGFR mutation in NSCLC

Increase in dimer formation limits the treatment efficacy in the EGFR receptor, which leads to drug resistance development. Compound JBJ-04-125-02, a mutant selective allosteric inhibitor of EGFR. It comprises of isoindolinone moiety at carbon-6, with a 5-indole substitution. JBJ-04-125-02 is reported to be more potent than EAI045 and inhibit the cell proliferation and EGFR L858R/T790M/C797S signalling at better efficacy. The compound expresses sub-nanomolar biochemical potency against EGFR L858R/T790M/C797S signalling in various assay conditions with IC_50_ value 0.26 nmol/L. The response rate (RR) is observed to be 62–71%, and progression-free survival (PFS) was 9.9–12.5, which is more efficacious than chemotherapy regimen [50].

JBJ-04-125-02 exhibits its action by binding to the allosteric pocket without displacing the αC-helix. The hydrogen bond is formed between the Phe856 and Glu865 of EGFR kinase protein. One *π*–*π* stack interaction with Phe723 in kinase P-loop. These are the main inhibitory sites of EGFR to stop cancerous growth. The half-life of JBJ-04-125-02 is recorded to be 3-h compared to other compounds in the series. The plasma concentration of 1.1 µmol/L is achieved with oral dosing and i.v dose showed a better effect. It is reported that JBJ-04-125-02 at 50 mg/kg and 100 mg/kg dose was able to inhibit auto-phosphorylation of EGFR, AKT and ERK1/2 when they are administered by i.v. [50].

JBJ-04-125-02, do not show any symptoms of weight loss or toxicity. But the oral bioavailability is poor, due to which over time if the patient continues to take the medication expects the drug accumulation in plasma. The drug shows an enhanced effect when combined with osimertinib [50]. Therefore, JBJ-04-125-02 compound has the potential to become the fourth-generation anti-NSCLC drug and shows more efficacy than chemotherapeutic agents due to its sub-nanomolar biochemical potency.

## Effect of DDC4002 against NSCLC

Compound DDC4002, (PDB i.d.: 6PID), have a 5,10-dihydro-11*H*-dibenzo [b,e] diazepine-11-one scaffold. It comprises of a seven-membered diazepinone ring, which is packed inward the α-C-helix. This compound has been reported to inhibit the L858R/T790M and L858R/T790M/C797S EGFR mutation and have IC_50_ of ~ 0 nM. The compound DDC4002, when interacting with EGFR allosteric site, leads to the extension of N-lobe leading to benzyl substitution between AMP and PNP, side-chain of K795, L788 and T790M, the gatekeeper-mutation in cells. This ultimately leads to inhibition of cell proliferation and inhibition of caspase-7 pathway. The reports also suggest that DDC4002 is more efficacious than compound EAI045. DDC4002 showed EGFR biochemical activity against L858, therefore, DDC4002 have the potential to become an anti-NSCLC agent and can solve the drug resistance development problem effectively [51].

## Small molecules targeting allosteric sites of EGFR

Caporuscio et al. [49], have reported small affinity molecules by low-cost, high throughput docking method. These compounds were identified from the Enamine Advanced Collection (2,50,000 compound library) and Life Chemicals collection (3,70,000 compound library). Smart substitution filter removed Pan-Assay Interference Compounds (PAINS) and induced fit docking leads to the identification of the top-10 compounds with good interaction to the allosteric site of EGFR. The compounds had IC_50_ ~ 14 µM. Among these compounds, the compound 8 reported the least IC_50_ or highest EGFR inhibitory activity (Table 2). This is due to (*R*)-enantiomer of eight hydrogen bond to Lys745 side chain. The nitrogen molecule in indole showed hydrogen bond formation with the Phe856 carbonyl group in the allosteric site of EGFR. The nitrophenylalanine of the group showed hydrogen bond interaction with Asp855 of the receptor [45]. The apolar moiety of these compounds showed Van der Wall interaction with Phe 723, Ala 747, Met 766, Leu 777, Leu 788 Ile 789, Met 790 and Phe 856. These main allosteric interaction sites were identified. All of the ten short-listed compounds showed hydrogen bond interaction of the (*S*)-enantiomer conformation of these compounds with the carbonyl group of Asp 855 [49].

## Future prospective

NSCLC is the leading cause of death among all oncology cases. EGFR is a potential target for developing drugs in NSCLC [52]. The effectiveness of EGFR TKIs used in targeted cancer treatment therapy is very often restricted owing to the development of acquired resistance, which is often triggered by fresh mutations in targeted kinases. EGFR TK inhibitors have become one of the most sophisticated NSCLC therapy models with kinase mutation. Treatment with current therapy for EGFR-mutated NSCLCs leads to drug resistance due to EGFR T790M mutation in 50–60% of instances. Although drugs of the third generation can overcome EGFR T790M, a fresh EGFR mutation, C797S, holds the effectiveness of these agents to a standstill. It is, therefore, the paramount requirement in this sector to find fresh agents to overcome EGFR T790M/C797S [46, 47, 49]. EGFR allosteric inhibitors such as EAI045 have the potential to inhibit aggressive tumor growth that harbour EGFR L858R/T790M/C797S in conjunction with cetuximab. This results in complex toxicity conditions and certainly escalate the clinical expenses significantly, in conjunction with cetuximab. Fourth-generation drugs require further studies so that they are suitable as a single agent for targeting EGFR Del-19/T79M/C797S. The fourth-generation anti-lung cancer therapy showed lesser laminations as compared to other therapy available for NSCLC [7, 47]. A recent review emphasize on optimised therapy of combined targeted therapy, immunotherapy, and chemotherapy. The authors have also described the non-availability of predictive markers in clinical and preclinical research on NSCLC [53]. In future, it can help to develop EAI045 with its potent EGFR inhibition property than EAI001 and its specificities for T790M. Further bench studies and biochemical optimization followed by clinical trials are necessary to confirm the efficacy of the fourth generation drugs in patients with advanced NSCLC. The discovery of the potent fourth-generation anti-NSCLC agents was possible because of the efficient, quick and cheap high-throughput screening methodologies followed using various in silico technology. The biological activity of the identified allosteric inhibitors of EGFR was confirmed through various in vitro and in vivo assays. Therefore, for quick identification of new drugs and to fast-track the research methodology, the use of advanced computer-technology along with the standardization of confirmatory biological assay is imperative. The future of drug discovery through analogue searches in various libraries of compounds and its synthetic effects can expedite the fourth generation NSCLC agents. This will also facilitate exploring the structure–activity relationships of new inhibitors and can yield compounds with an improved activity profile with respect to the hit compounds.
